# Hierarchical Modeling of Patient and Physician Determinants of Blood Pressure Outcomes in Hypertensive Patients with and without Diabetes: Pooled Analysis of Six Observational Valsartan Studies with 15,282 Evaluable Patients

**DOI:** 10.1155/2017/9842450

**Published:** 2017-09-25

**Authors:** Noha Ashy, Thanh-Nga Nguyen, Kris Denhaerynck, Mahdi Gharaibeh, Abdulaziz Alhossan, Stefaan Vancayzeele, Heidi Brié, Ann Aerts, Karen MacDonald, Ivo Abraham

**Affiliations:** ^1^College of Pharmacy, King Abdulaziz University, P.O. Box 80200, Jeddah 21589, Saudi Arabia; ^2^Center for Health Outcomes and PharmacoEconomic Research, University of Arizona, 1295 N Martin, Tucson, AZ 85721, USA; ^3^College of Pharmacy, University of Wyoming, 1000 E University Ave, Laramie, WY 82071, USA; ^4^Matrix45, 6159 W Sunset Rd, Tucson, AZ, USA; ^5^Institute of Nursing Science, University of Basel, Bernoullistrasse 29, 4001 Basel, Switzerland; ^6^Cardiovascular Research Institute Basel, University Hospital Basel, Spitalstrasse 2, 4056 Basel, Switzerland; ^7^College of Pharmacy, King Saud University, P.O. Box 89885, Riyadh 11692, Saudi Arabia; ^8^Novartis Pharma, Medialaan 40, 1800 Vilvoorde, Belgium; ^9^Novartis Foundation, Novartis Campus, Forum 1-3.97, 4002 Basel, Switzerland; ^10^Department of Pharmacy Practice and Science, College of Pharmacy, University of Arizona, 1295 N Martin, Tucson, AZ 85721, USA; ^11^Department of Family and Community Medicine, College of Medicine-Tucson, University of Arizona, 1295 N Martin, Tucson, AZ 85721, USA

## Abstract

We pooled data from 6 valsartan-related studies including 3,658 diabetic and 11,624 nondiabetic patients to evaluate blood pressure (BP) outcomes after approximately 90 days of second- or later-line valsartan treatment. Hierarchical linear and logistic regressions were applied to identify determinants of BP outcomes. Similar reductions in BP values and similar BP control rates were achieved in both groups after approximately 90 days of therapy. The modeling analyses identified several common and different patient- and physician-related determinants of BP outcomes for both groups, many of which are modifiable or clinically manageable. Through varying in terms of association and influence between the diabetic and nondiabetic groups, patient-related determinants included age, BP at diagnosis of hypertension, risk factors, valsartan regimen, concomitant antihypertensive treatment, and adherence; and physician-related determinants included gender, years in practice, and hypertension management. In summary, in both diabetic and nondiabetic patients, the use of valsartan-centric treatment regimens in second- or later-line antihypertensive treatment is associated with significant reductions in BP level and improvement in BP control. The determinants identified in modeling provide guidance to clinicians in the common and differential management of hypertension in diabetic and nondiabetic patients.

## 1. Introduction

Hypertension and glucose intolerance are highly associated chronic conditions. Over two-thirds of diabetic patients have elevated blood pressure [1]. Diabetes and hypertension have long been known to be major risk factors for cardiovascular mortality and morbidity [2]. Diabetes increases the risk of cardiovascular disease twofold to threefold, while high blood pressure (BP) is associated with a 72% rise in all-cause mortality risk and a 57% rise of cardiovascular events in diabetic individuals [3, 4].

For long, the recommended systolic blood pressure (SBP) and diastolic blood pressure (DBP) targets were <140 mmHg and <90 mmHg for nondiabetic patients but <130 mmHg and <80 mmHg for diabetic patients. The most recent European [5] and North American [6] guidelines now recommend SBP < 140 mmHg and DBP < 90 mmHg in the general adult population for both diabetic and nondiabetic patients. It is more difficult to achieve blood pressure control in diabetic patients [5], but the revised targets for these patients have resulted in nominally higher BP control rates in this population.

We have conducted seven observational studies on real-world practice patterns and associated outcomes in patients with hypertension treated for approximately 90 days (hereafter “90 days”) with valsartan (Novartis, Basel, Switzerland), an angiotensin receptor blocker [7]. All patients were treated by general practitioners (GPs) in Belgium in second- or later-line antihypertensive therapy because prior antihypertensive treatment failed or was not tolerated. Because of similarities in design and data model, in particular the assessment of medication adherence, we pooled six of these studies to yield a data set of 15,282 evaluable patients recruited by 2,832 general practitioners (GPs) (Table 1). Of these patients, 3,658 (23.9%) had a diagnosis of either type 1 or type 2 diabetes mellitus.

Lowering blood pressure and especially achieving blood pressure control are more difficult in diabetic than in nondiabetic patients, as both randomized controlled trials and observational studies have shown [4–6]. There is a continued need to validate the known determinants of these differential outcomes and to identify other determinants, especially in the real-world setting of routine clinical practice. Similarly, there is a need to examine which determinants of outcome are common (and to what extent) among diabetic and nondiabetic patients and where these two cohorts differ (and to what extent) in terms of determinants. By necessity, randomized controlled trials evaluate treatments under conditions of relative homogeneity in patients, settings, clinicians, and treatment protocols. In contrast, studies in the real-world setting assess treatments under conditions of heterogeneity in patients, settings, clinicians, and treatment approaches.

In the comparative analysis reported here, we aimed (1) to evaluate blood pressure values (in mmHg) and blood pressure control rates (in %) observed after 90 days of treatment with a valsartan-centric regimen and (2) to identify patient-related and clinician-related determinants of these blood pressure outcomes using hierarchical linear and logistic regressions. Implicit to the choice for hierarchical models of statistical analysis was the hypothesis, evaluated and confirmed in each of the constituent studies in this pooled analysis; that part of the variance in BP values at the end of the observation period could be attributed to a class effect for the treating physician. Also, our aim was to evaluate similarities and differences in the determinants of blood pressure outcomes between diabetic and nondiabetic patients as strata and therefore we opted for our analysis method rather than evaluating models of determinants and using diabetic status as a cofactor. The studies were conducted before the change in guidelines concerning BP targets from <140 mmHg/90 mmHg for patients in general but <130 mmHg/80 mmHg for diabetic patients. However, we adopted the recent <140 mmHg/<90 mmHg targets as the threshold for BP control.

## 2. Methods

The methodology common to all of the studies included in this pooled analysis has been described in detail elsewhere [7]. We summarize key aspects here.

### 2.1. Design, Subjects, and Data

All six studies were prospective, multicenter, pharmacoepidemiological studies of hypertensive patients in the setting of routine “real-world” clinical practice involving hypertensive patients whose GPs prescribed a valsartan formulation, in monotherapy or combination therapy, as second- or later-line antihypertensive therapy because prior treatment failed to achieve the intended therapeutic benefit or was not tolerated. Patients BP was measured when valsartan therapy was initiated and again after approximately 90 days of therapy. The latter visit occurred as physician and patient schedules permitted in routine clinical practice and may not have been exactly 90 days (hereafter referred to as “90 days”). The decision to prescribe valsartan was made by the prescribing physician per best clinical judgment.

Note that the one study excluded from this pooled analysis did not collect data using the Basel Assessment of Adherence Scale and in particular the item asking patients whether they had missed any doses in the preceding four weeks. Consistently, previous analyses have shown adherence to be a significant determinant of blood pressure outcomes and this single item has been shown to be highly predictive of outcomes [8]. Hence, this one study was not included in this pooled analysis.

As the constituent studies were observational in design, all data were recorded as available from routine clinical practice. There were no mandatory tests or other assessments. The common data model for this study has been described extensively elsewhere [7]. In addition to patient-related variables, the data model also included several physician-related variables.

A subject was considered evaluable if SBP and DBP values (mmHg) were available at both baseline and 90 days. Patients were stratified as diabetic or nondiabetic based on whether a diabetes diagnosis was recorded by their treating physician.

We treated diabetic status as a stratification variable and hence distinguished between the strata of diabetic and nondiabetic patients. This was driven by our aim to evaluate not only blood pressure outcomes but also especially the similarities and differences in the determinants of blood pressure outcomes between diabetic and nondiabetic patients as strata. Hence, we did not use diabetic status as a cofactor in an omnibus model and opted for stratified analyses.

### 2.2. Statistical Analysis

We assumed that the patients recruited by a given GP had the commonality of being treated by the same GP and thus within the knowledge, experience, treatment preferences, and practice patterns of this GP. This violates the statistical assumption of independence of observations, which can be corrected by hierarchical (multilevel or mixed-effects) modeling. In this modeling, the variance observed in an outcome variable (in this case, blood pressure outcomes) is partitioned between the treating physicians and the patients being treated. This enables differentiation of the proportion of variance attributable to, in this case, the treating physician as a class effect (expressed in the metric of intraclass correlation coefficient [ICC]) and the residual variance to the patients being treated [9]. Conditional two-level (physician level and patient level) mean effect mixed models analysis was performed to determine mean SBP and DBP, quantify the treatment effect after 90 days, and, using backward manual elimination, identify physician-level and patient- level determinants of BP values in the diabetic and nondiabetic groups. Adjusted logistic regression model with backward manual elimination was used to identify predictors of uncontrolled SBP (≥140 mm Hg), DBP (≥90 mm Hg), and combined SBP/DBP in the diabetic and nondiabetic cohorts. Backward manual elimination was used to identify independent variables included in the logistic regression model while assuring clinical relevance over statistical prioritization. Because of the relative lack of uniformity in goodness-of-fit metrics for hierarchical and logistic regression modeling, we chose not to rely on prespecified metrics for two reasons. First, we chose to evaluate the goodness-of-fit metrics for hierarchical linear and hierarchical logistic modeling. Second, we monitored for the precision of estimates generated in the modeling analyses.

The constituent studies differed to some extent in the potency of the valsartan formulations that were evaluated (Table 1). Hence studies were considered a proxy for the formulation(s) being evaluated and included in the analyses as a potential patient-level determinant. Any study effects retained in the modeling were identified by the study's name (Table 1).

All tests were two-tailed and a *P* value less than 0.05 was considered as statistically significant. Statistical analyses were performed using SAS v.9.4 (SAS Institute, Cary, NC, USA).

## 3. Results

### 3.1. Patients

Of the 15,282 patients in the evaluable sample, 3,658 (23.9%) were diabetic and 11,624 (76.1%) were nondiabetic.  Table 2 summarizes patient demographics, cardiovascular risk factors and history, and antihypertensive treatment patterns at baseline (initiation of valsartan treatment) for the nondiabetic and diabetic cohorts. There were no statistically significant differences between both cohorts except for higher proteinuria and diabetic nephropathy rates in the diabetic group.

### 3.2. BP Values and Control Rates at 90 Days Compared to Baseline

In both the diabetic and nondiabetic groups, SBP and DBP values (mmHg) decreased significantly over the course of treatment (all *P* < 0.001) (Table 3). The nominal differences in recorded SBP and DBP reductions were statistically similar (all *P* = n.s.). Similarly, SBP, DBP, and combined SBP/DBP control rates improved significantly during the 90-day study period (all *P* < 0.001). The nominal differences in control rates were statistically similar as well (all *P* = n.s.).

### 3.3. Linear Modeling of SBP Values

In the diabetic group, from an intercept of 111.02 mmHg, SBP values increased as a function of the SBP recorded at the time of the hypertension diagnosis, total cholesterol levels at start of the study, higher valsartan and hydrochlorothiazide (HCTZ) doses prescribed (including a study effect for the PREVIEW study), and the need for concomitant beta-blocker therapy. At the physician level, being treated by a male GP was associated with higher SBP values (Table 4). In contrast, patient adherence had a mitigating effect on SBP as did, perhaps counter-intuitively, the presence of cardiovascular disease. The ICC for the SBP model in the diabetic cohort was 0.25.

In the nondiabetic group, from an intercept of 105.79 mmHg, SBP values rose as a function of patient age, the SBP recorded at the time the hypertension diagnosis was made, total cholesterol levels, body mass index (BMI), higher valsartan and hydrochlorothiazide (HCTZ) doses prescribed, and the need for concomitant beta-blocker therapy. At the physician level, years in practice and male gender were associated with higher SBP values (Table 4). Patient adherence had a mitigating effect as did, perhaps counter-intuitively, the presence of renal impairment and cardiovascular disease. The ICC for the SBP model in the nondiabetic group was 0.23.

### 3.4. Linear Modeling of DBP Values

In the diabetic group, from an intercept of 74.14 mmHg, DBP values rose as a function of the DBP recorded at the time of the hypertension diagnosis, total cholesterol levels at start of the study, and higher valsartan and hydrochlorothiazide (HCTZ) doses (including a study effect for the PREVIEW study). At the physician level, years the GP had been in practice were associated with higher DBP readings (Table 4). Alternately, patient age and patient adherence had a mitigating effect on DBP as did, perhaps counter-intuitively, the presence of cardiovascular disease and (per the study effect observed for the IMPROVE study) higher valsartan and HCTZ doses when in single-pill combinations with valsartan. At the physician level, the time typically taken with a newly diagnosed hypertension patient in the first visit also had a mitigating effect on DBP. The ICC for the SP model in the diabetic group was 0.24.

In the nondiabetic group, from an intercept of 69.26 mmHg, DBP values increased as a function of the DBP recorded at the time of hypertension diagnosis, total cholesterol levels, body mass index (BMI), and higher valsartan and hydrochlorothiazide (HCTZ) doses prescribed. At the physician level, male gender was associated with higher DBP values (Table 4). Patient age had a mitigating effect as did the number of hypertensive patients the treating GP had seen in the preceding year. The study effect for the INSIST study indicated the therapeutic benefit of the single-pill combination of 160 mg valsartan and 25 mg HCTZ. The ICC for the SBP model in the nondiabetic cohort was 0.24.

### 3.5. Logistic Modeling of SBP Control Rates

In the diabetic cohort, the odds of controlled SBP at 90 days decreased as a function of total cholesterol levels and the need for higher valsartan and HCTZ doses (Table 5). The odds of controlled SBP at 90 days in this cohort also decreased if patients were started on low valsartan and/or HCTZ doses (as indicated by the study effect for the PREVIEW study) (Table 5). Perhaps counter-intuitively, the odds of controlled SBP in the diabetic group rose if patients had cardiovascular disease.

In the nondiabetic cohort, the likelihood of controlled SBP at 90 days increased if the patient was adherent and (per the INSIST study effect) was treated with the single-pill combination of 160 mg valsartan and 25 mg HCTZ (Table 5). Impairing the probability of controlled SBP in this group were patient age, the SBP recorded at the time of diagnosis, renal impairment, total cholesterol levels, the need for higher valsartan and HCTZ doses, and, at the physician level, the years the treating GP had been in practice.

### 3.6. Logistic Modeling of DBP Control Rates

In the diabetic group, the likelihood of controlled DBP at 90 days increased as a function of patient age, patient adherence, and treatment with proportionately more potent valsartan and HCTZ formulations (as indicated by the study effect for the IMPROVE study) (Table 5). The odds of controlled DBP at 90 days decreased as a function of the DBP recorded at the time the hypertension diagnosis was made, total cholesterol levels, and the need for higher HCTZ dose at the start of the study.

In the nondiabetic cohort, the odds of controlled SBP at 90 days increased with age and if the patient was adherent and (per the INSIST study effect) was treated with the single-pill combination of 160 mg valsartan and 25 mg HCTZ and, counter-intuitively, had cardiovascular disease (Table 5). At the physician level, the number of hypertension patients seen in the prior year also increased the odds of controlled DBP at 90 days. Decreasing the likelihood of controlled DBP in this group were the DBP recorded at the time of diagnosis, total cholesterol levels, and the need for higher valsartan and HCTZ doses.

### 3.7. Logistic Modeling of Combined SBP and DBP Control Rates

In the diabetic group, the odds of having both SBP and DBP controlled at 90 days were a function of patient adherence and the counterintuitive finding of having cardiovascular disease (Table 5). The odds of controlled SBP/DBP at 90 days decreased as a function of the SBP level at the time the hypertension diagnosis was made, total cholesterol levels, and the need for higher valsartan and HCTZ doses at the start of the study (as also indicated by the study effect for the PREVIEW study).

In the nondiabetic cohort, the likelihood of controlled SBP/DBP increased with patient adherence and treatment with potent valsartan and HCTZ formulations (per the INSIST study effect) (Table 5). Lowering the odds of controlled SBP/DBP in this group were the SBP at the time of hypertension diagnosis, total cholesterol levels, the need for higher valsartan and HCTZ doses (as also indicated by the study effect for the PREVIEW study), and, at the physician level, the number of years the GP had been in practice.

## 4. Discussion

The first principal finding of this pooled analysis comparing 3,658 patients with diabetes and 11,624 patients without diabetes treated with valsartan (second or later line) is that similar reductions in BP values and similar BP control rates can be achieved in both groups. However, the patient- and physician-level determinants of these outcomes differ between these groups. On average, in diabetic patients, SBP was reduced by 17.1 mmHg and DBP by 8.8 mmHg, compared to 18.9 mmHg and 9.9 mmHg in nondiabetic patients. In both groups, at follow-up, eight out of ten patients had controlled DBP, half of the patients had controlled SBP, and slightly less than half had controlled SBP/DBP. Thus, in our analysis, diabetic patients showed similar decreases in blood pressure values and achieved similar blood pressure control rates as nondiabetic patients. These findings are remarkable for diabetic patients because of the known difficulty in lowering BP and achieving BP control in this population.

The second principal finding is that the diabetic and nondiabetic strata have some common but also some unique determinants of blood pressure outcomes. Where the same determinants were observed, the strata often differed in the impact and weight of a given determinant on blood pressure outcomes.  Table 6 presents a conceptual summary of the determinants by strata.

Summarizing key results, higher blood pressure at the time of diagnosis, elevated total cholesterol, and higher valsartan and concomitant HCTZ doses were associated with an undesired effect on blood pressure outcomes in both strata of patients. However, generally, these effects were more pronounced in diabetic patients. In contrast, adherence had a desired effect on blood pressure outcomes in both strata but especially among diabetic patients.

There were also some isolated effects, desired and undesired, for diabetic and nondiabetic patients. The need for concomitant beta-blocker therapy was associated with worse BP outcomes. There was also a trend of poorer outcomes among patients whose GP had been in practice longer and, we can presume, may therefore also have been older. In both diabetic and nondiabetic patients, age was associated with better DBP outcomes. In contrast, among nondiabetic patients, age was associated with poorer SBP outcomes. BMI had an undesired effect on blood pressure values in nondiabetic patients.

At the physician level, diabetic patients benefitted from their physicians spending more time with them when they were diagnosed with hypertension. Nondiabetic patients, in contrast, showed better BP outcomes if their GPs had a large volume of hypertensive patients. For both diabetic and nondiabetic patients, there was an association between male physician gender and poorer blood pressure outcomes. This may be tied also to the observed association of poorer outcomes in patients followed by older physicians.

We determined BP control rates using the most recent European [5] and North American [6] criteria of SBP < 140 mmHg and DBP < 90 mmHg. However, if the criteria for diabetics at the time of the conduct of the studies were applied, that is, SBP < 130 mmHg and DBP < 80 mmHg, control rates would have been 17.6% for SBP, 25.5% for DBP, and 9.5% for combined SBP/DBP. This suggests that one-third of diabetic patients had SBP between 130 mmHg and 140 mmHg and over half had DBP between 80 mmHg and 90 mmHg at follow-up (data not reported). The encouraging BP control rates reported here are directly related to the recent change in BP criteria.

At the time of the studies, valsartan was indicated for second- or later-line treatment if prior-line treatment did not achieve the intended therapeutic benefit or was not tolerated. Our analyses yielded a perhaps paradoxical finding: higher doses of valsartan and HCTZ, and perhaps the addition of a beta-blocker, were associated with poorer BP outcomes in both diabetic and nondiabetic patients. Keeping in mind that the studies were in the GP setting, this may reflect that these clinicians were trying to bring this previously uncontrolled BP under control but may not have yet achieved this for many patients during the 90-day observation period. Also, note that being a subject in the IMPROVE (valsartan 80 mg to 160 mg, with or without HCTZ 12.5 mg to 25 mg) and PERSIST (valsartan/HCTZ 160 mg/25 mg) studies was associated with better BP outcomes to which can be added the EXCELLENT study (valsartan/amlodipine 80–160 mg/5–10 mg) as this was the reference study. These two trends in the data may reflect the real-world fact that, despite being treated with combination therapies, some patients may still not achieve the desired BP outcomes, while other patients receiving the same or different combination therapies do show beneficial BP outcomes. The issue may not be whether or not combination therapies are indicated. Rather, there may be a need to assess which combinations of agents are effective for which profiles of patients. Future studies, in first instance in diabetic patients, are needed to evaluate which antihypertensive combination therapies will yield for specific patients the greater reductions in BP and larger proportions of patients with controlled BP. This need for additional investigations, especially in diabetic patients, is also supported by the finding that lower cholesterol levels were associated with better outcomes, underscoring the need for lipid-lowering therapy and lifestyle modifications. This also applies to the nondiabetic group. Moreover, in this cohort, BMI was retained as a determinant in some models, indicating the importance of weight management in this population.

Our findings emphasize the importance of adherence in patients in general but certainly in diabetic patients. Adherence was associated with lower SBP and DBP values and higher odds of achieving BP control. In an analysis of this same pooled data set comparing adherent and nonadherent patients, BP values and BP control rates were consistently better among adherent patients [10]. Interestingly, this analysis revealed that adherence to antihypertensive regimens may be a function of prior treatment-line failure, severity of illness, and patients experiencing (major) health problems related to their hypertension and/or diabetes. Particularly the latter may motivate patients to change their medication behavior. Noteworthy also was the fact that medication adherence tended to be better if the treating GPs were female.

Focusing specifically on our findings for diabetic patients, previous studies have shown that tight BP control in diabetic individuals is associated with better morbidity and mortality outcomes. In the UK Prospective Diabetes Study (UKPDS), diabetic patients with high BP were randomly assigned to receive either tight (<150/85 mm Hg) or less tight blood pressure control (<180/105 mm Hg) for a median follow-up of 8.4 years. Compared to the less intensively treated group and taking into account the fact that these targets are well above current guidelines, tight BP control was associated with a significant reduction in the diabetes endpoint and in the diabetes-related death, stroke, and microvascular endpoints [11, 12]. These findings were supported by a retrospective analysis of the diabetes subgroup (*n* = 1501) of the Hypertension Optimal Treatment (HOT) trial, where DBP ≤ 80 mmHg was associated with a significant reduction in major cardiovascular events compared to those assigned to the goal of ≤90 mmHg [13, 14].

Some of our findings were counter-intuitive; specifically, cardiovascular disease (defined as having had a myocardial infarct or having coronary artery disease) and renal impairment were associated with better BP outcomes. As we have argued before [7], the retention of these variables in the models may be a proxy for greater clinician vigilance in managing hypertension in patients with these conditions. In fact, diabetes itself may trigger greater clinician vigilance. These seemingly counter-intuitive findings may also suggest that clinicians adhere to guidelines and specifically those pertaining to risks associated with comorbidities. However, this needs to be investigated further because some of the studies in our pooled data set have shown that GPs tend not to know guidelines well and do not tend to practice in accordance with these guidelines [7]. Our findings also accentuate the importance of focusing on the hypertension, diabetes, and chronic kidney disease triangle and its consequences rather than each condition separately [15].

BP outcomes are determined, at least in part, by the treating physician, in the case of our data, the GP. The intraclass correlation coefficients confirm that about a quarter of the variation in BP readings at follow-up may be accounted for by a clinician class effect [7]. Further, and this applied in particular to nondiabetic patients, outcomes tended to be relatively poorer if the GP was male and older (as indicated by years in practice) [16]. While older practitioners have the benefit of accumulated experience, they may be less likely to intensify treatment and thus exhibit more therapeutic inertia [17, 18]. On the other hand, a GP's volume of hypertensive patients was associated with better BP outcomes as was the time a GP typically spends with a newly diagnosed hypertensive patient. Lastly, one area that merits clinical consideration concerns the early detection of hypertension. Consistently, higher SBP and DBP at the time the diagnosis of hypertension were associated with poorer BP outcomes. More timely diagnosis, which requires both clinical attention to and screening of patients at risk for hypertension, is indicated.

Our pooled analyses have limitations. All six studies were noncontrolled observational studies, not randomized controlled trials. On the other hand, the fact that the studies shared a nearly identical methodology and yielded a pooled sample in excess of 15,000 patients emphasizes the strength of the observed associations in the diabetic and nondiabetic groups. The groups were unbalanced in size, though they mirrored the distribution of diabetic and nondiabetic patients with hypertension. This could have affected the intercept and other estimates and their relative precision. Although not a guarantee, the large sample and the variation in outcomes observed should have buffered against this, thus foregoing the need to apply corrections to the intercept. We were interested in a comparative analysis of diabetic and nondiabetic patients; hence we did not construct a model that uses diabetic status as a cofactor. Relatively few physician-specific determinants were retained in the models, despite the finding that about a quarter of the variance in BP readings was attributable to a physician class effect. The six constituent studies involved valsartan-centric regimens and the findings may not be generalizable to other regimens. Applicable though not specific to our study, diurnal blood pressure variation could have affected measurements, though this would be difficult to control or account for in observational studies of real-world practice patterns and outcomes. The lack of generally agreed upon goodness-of-fit metrics for hierarchical linear and logistic regressions may have lent some subjectivity to the adoption of models.

## 5. Conclusion

In both diabetic and nondiabetic patients, the use of valsartan-centric treatment regimens in second- or later-line antihypertensive treatment was associated with significant reductions in SBP and DBP values and with significant increases in SBP, DBP, and combined SBP/DBP control rates. Common and unique patient- and physician-related determinants of these outcomes were identified. These provide guidance to the management of hypertension in diabetic and nondiabetic patients treated with valsartan: the need for timely diagnosis of hypertension; the importance of aggressive treatment and avoiding therapeutic inertia; attention to such factors as BMI, lipid levels, comorbidities, adverse events, and relevant medical history; and assessing and promoting patient adherence. On the clinical side, clinicians are advised to spend more time with diabetic patients when they are first diagnosed with hypertension and to gain extensive clinical experience in managing hypertension. Valsartan proved to be both effective and safe in reducing blood pressure in adults with essential hypertension.

## Figures and Tables

**Table 1:** Studies included in pooled analysis.

	PREVIEW	IMPROVE	INSIST	eNOVA	*B*SCORE	EXCELLENT	Total
*Study characteristics*							
Year initiated	2004	2004	2006	2006	2008	2008	
Diabetic	627	781	225	253	815	957	3658
Nondiabetic	2424	2818	470	701	2622	2589	11624
Number of physicians	504	684	308	284	354	698	2832

	PREVIEW	IMPROVE	INSIST	eNOVA	*B*SCORE	EXCELLENT	Weighted average

*Patient characteristics*							
Age, y, mean (±SD)	63.4 ± 11.9	63.2 ± 12.3	63.6 ± 12.0	64.0 ± 11.4	63.8 ± 12.0	63.8 ± 11.7	63.6 ± 12.0
Male gender (%)	47.7	48.7	48.5	49.0	52.3	53.9	50.5
Valsartan formulation							
80 mg	✓	✓		✓	✓		
160 mg	✓	✓		✓	✓		
80/12.5 mg HCTZ	✓	✓		✓	✓		
160/12.5 mg HCTZ		✓		✓	✓		
160/25 mg HCTZ		✓	✓		✓		
80/5 mg amlodipine						✓	
160/5 mg amlodipine						✓	
160/10 mg amlodipine						✓	

HCTZ: hydrochlorothiazide; SD: standard deviation.

**Table 2:** Patient characteristics by diabetes status.

	Diabetic	Nondiabetic	*P*
*Demographics*			
Age, y, (M ± SD)	65.44 ± 10.38	63.10 ± 12.24	n.s.
Male gender (%)	51.18	51.66	n.s.

*Cardiovascular risk factors and history*			
Smoking (%)	22.03	25.64	n.s.
Claudication (%)	9.44	5.59	n.s.
Adherence (%)	71.29	73.26	n.s.
Total cholesterol, mg/dl, mean (±SD)	205.9 ± 43.3	214.7 ± 39.0	n.s.
Fasting LDL, mg/dl, mean (±SD)	111.3 ± 45.0	119.5 ± 42.3	n.s.
Fasting HDL, mg/dl, mean (±SD)	67.6 ± 34.1	68.4 ± 33.9	n.s.
Microalbuminuria (%)	24.80	4.16	n.s.
Proteinuria (%)	11.95	2.00	<0.010
Renal impairment (creatinine > 1.5 mg/dl) (%)	6.92	2.70	n.s.
Diabetic nephropathy (%)	12.13	0.42	<0.003
Amputation (%)	0.66	0.14	n.s.
Angina (%)	20.05	13.12	n.s.
Transient ischemic attacks (%)	9.44	7.00	n.s.
Peripheral bypass/stent (%)	7.99	5.19	n.s.
Coronary revascularization (%)	12.53	7.68	n.s.
Cerebrovascular accident (ischemic) (%)	6.99	5.10	n.s.
Myocardial infarct (%)	11.27	7.62	n.s.
Left ventricular hypertrophy (%)	19.89	12.40	n.s.
Congestive heart failure (%)	6.70	3.73	n.s.
Cerebrovascular accident (hemorrhagic) (%)	1.15	0.76	n.s.

*Antihypertensive treatment patterns*			
Valsartan 80 mg (%)	4.93	7.15	n.s.
Valsartan, 160 mg (%)	35.14	40.70	
Valsartan 80/12.5 mg HCTZ (%)	5.77	6.78	
Valsartan 160/12.5 mg HCTZ (%)	16.06	14.89	
Valsartan 160/25 mg HCTZ (%)	11.90	8.20	
Valsartan 80/5 mg amlodipine (%)	2.24	2.69	
Valsartan 160/5 mg amlodipine (%)	15.96	15.38	
Concomitant diuretic (%)	28.70	22.29	n.s.
Concomitant alpha-blocker (%)	4.76	2.06	n.s.
Concomitant beta-blocker (%)	37.07	32.79	n.s.
Concomitant calcium antagonist (%)	48.80	37.01	n.s.
Concomitant ACE inhibitor (%)	6.45	2.48	n.s.

M: mean; SD: standard deviation; n.s.: nonsignificant; ACE: angiotensin-converting enzyme.

**Table 3:** SBP and DBP at baseline and 90 days by diabetes status.

	Diabetic				Nondiabetic			
	Baseline	90 days	Δ	*P*	Baseline	90 days	Δ	*P*
*Blood pressure (mmHg)*								
SBP (M ± SD)	155.0 ± 15.2	137.9 ± 12.5	−17.1	<0.001	156.1 ± 15.5	137.2 ± 11.7	−18.9	<0.001
DBP (M ± SD)	90.4 ± 9.7	81.6 ± 7.6	−8.8	<0.001	91.8 ± 9.5	81.9 ± 7.5	−9.9	<0.001

	140/90 mmHg				140/90 mmHg			

*Blood pressure control (%)*								
SBP	7.8	51.2	43.4	<0.001	7.9	52.5	44.6	<0.001
DBP	34.6	81.2	46.6	<0.001	29.0	80.6	51.6	<0.001
SBP/DBP	6.3	48.0	41.7	<0.001	6.1	48.8	42.6	<0.001

SBP: systolic blood pressure; DBP: diastolic blood pressure; SBP/DBP: combined SBP & DBP; M: mean; SD: standard deviation.

**Table 4:** Hierarchical linear modeling of SBP and DBP at 90 days by diabetes status.

	Diabetic				Nondiabetic			
	Estimate	SE	*t*	*P*	Estimate	SE	*t*	*P*
SBP								

Intercept	111.02	2.8471	38.99	<0.001	105.79	2.0141	52.53	<0.001
**Patient determinants**								
Age (per 1 year)					0.0312	0.0112	2.78	0.006
SBP at diagnosis of HT (per 1 mmHg)	0.1097	0.0137	7.99	<0.001	0.1262	0.0089	14.20	<0.001
Renal impairment					−2.0430	0.7882	−2.59	0.010
Cardiovascular disease (MI & coronary)	−1.9713	0.6510	−3.15	0.002	−1.7733	0.4235	−4.19	<0.001
Total cholesterol (per 1 mg/dl)	0.0168	0.0054	3.12	0.002	0.0145	0.0035	4.12	<0.001
Body mass index (per 1 kg/m^2^)					0.0407	0.0151	2.69	0.007
Valsartan dose (0/80/160 mg)	1.8257	0.5433	3.36	0.001	1.1446	0.3190	3.59	<0.001
Hydrochlorothiazide (0/12.5/25 mg)	2.3898	0.5253	4.55	<0.001	3.0290	0.3188	9.50	<0.001
Concomitant beta-blocker	1.3436	0.4913	2.73	0.006	0.9209	0.2962	3.11	0.002
Adherence	−2.3483	0.5105	−4.60	<0.001	−2.1193	0.2969	−7.14	<0.001
Study: PREVIEW^¥^	3.3340	0.5693	5.86	<0.001				
**Physician determinants**								
Years in practice (per 1 year)					0.0794	0.0217	3.67	<0.001
Male gender	2.0623	0.7377	2.80	0.005	1.1902	0.5645	2.11	0.035
HT patients seen in past year (per 1 patient)					−0.0013	0.0006	−2.10	0.036
*Intraclass correlation coefficient*	0.25				0.23			

DBP								

Intercept	74.14	1.9590	37.85	<0.001	69.26	1.2184	56.84	<0.001
**Patient determinants**								
Age (per 1 year)	−0.0580	0.01337	−4.33	<0.001	−0.0363	0.0069	−5.23	<0.001
DBP at diagnosis of HT (per 1 mmHg)	0.0964	0.01304	7.40	<0.001	0.1139	0.0084	13.54	<0.001
Cardiovascular disease (MI & coronary)	−0.7409	0.3668	−2.02	0.044				
Total cholesterol (per 1 mg/dl)	0.0075	0.00323	2.33	0.020	0.0091	0.0022	4.17	<0.001
Body mass index (per 1 kg/m^2^)					0.0249	0.0097	2.55	0.011
Valsartan dose (0/80/160 mg)	0.9529	0.3214	2.97	0.003	0.3616	0.1969	1.84	0.066
Hydrochlorothiazide (0/12.5/25 mg)	1.2184	0.3194	3.81	<0.001	1.5947	0.2000	7.97	<0.001
Adherence	−1.5461	0.3037	−5.09	<0.001				
Study: IMPROVE^¥^	−1.3811	0.5008	−2.76	0.006				
Study: INSIST^¥^					−2.7437	0.6520	−4.21	<0.001
Study: PREVIEW^¥^	1.0204	0.4858	2.10	0.036				
**Physician determinants**								
Years in practice (per 1 year)	0.0499	0.01797	2.78	0.006				
Male gender					0.7676	0.3452	2.22	0.026
HT patients seen in past year (per 1 patient)					−0.0009	0.0004	−2.20	0.028
Duration of visit with newly diagnosed patient	−0.0636	0.02561	−2.48	0.013				
*Intraclass correlation coefficient*	0.24				0.24			

SBP: systolic blood pressure; DBP: diastolic blood pressure; HT: hypertension; MI: myocardial infarct; SE: standard error. ^¥^Reference study: EXCELLENT.

**Table 5:** Logistic regression modeling of controlled 90-day BP by diabetes status (140/90 mmHg).

	Diabetic		Nondiabetic	
	OR (95% CI)	*P*	OR (95% CI)	*P*
*SBP* control at 90 days				
**Patient determinants**				
Age (per 1 year)			0.996 (0.992–0.999)	0.022
SBP at diagnosis of HT (per 1 mmHg)	0.986 (0.981–0.991)	<0.001	0.980 (0.977–0.983)	<0.001
Adherence			1.443 (1.301–1.601)	<0.001
Renal impairment			0.696 (0.507–0.954)	0.024
Total cholesterol (per 1 mg/dL)	0.998 (0.981–1.000)	0.033	0.997 (0.996–0.999)	<0.001
Cardiovascular disease (MI & coronary)	1.300 (1.055–1.601)	0.014		
Valsartan dose (0/80/160 mg)	0.754 (0.632–0.900)	0.002	0.814 (0.731–0.907)	<0.001
HCTZ dose (0/12.5/25 mg)	0.770 (0.646–0.918)	0.004	0.672 (0.601–0.751)	<0.001
Study: PREVIEW^¥^	0.568 (0.438–0.737)	<0.001	0.699 (0.590–0.826)	<0.001
Study: INSIST^¥^			1.670 (1.196–2.332)	0.003
**Physician determinants**				
Years in practice (per 1 year)			0.988 (0.981–0.994)	<0.001

*DBP* control at 90 days				
**Patient determinants**				
Age (per 1 year)	1.015 (1.005–1.024)	0.002	1.007 (1.003–1.012)	0.003
DBP at diagnosis of HT (per 1 mmHg)	0.969 (0.956–0.979)	<0.001	0.965 (0.959–0.971)	<0.001
Adherence	1.348 (1.096–1.659)	0.005	1.443 (1.270–1.640)	<0.001
Cardiovascular disease (MI & coronary)			1.238 (1.016–1.510)	0.034
Total cholesterol (per 1 mg/dL)	0.998 (0.995–1.000)	0.020	0.998 (0.997–1.000)	0.0119
Valsartan dose (0/80/160 mg)			0.845 (0.734–0.972)	0.019
HCTZ dose (0/12.5/25 mg)	0.669 (0.541–0.827)	<0.001	0.650 (0.566–0.748)	<0.001
Study: INSIST^¥^			1.941 (1.248–3.019)	0.003
Study: IMPROVE^¥^	1.761 (1.230–2.519)	0.002	1.347 (1.055–1.719)	0.017
**Physician determinants**				
HT patients seen in past year(per 1 patient)			1.000 (1.000–1.001)	0.0063

*SBP/DBP* control at 90 days				
**Patient determinants**				
SBP at diagnosis of HT (per 1 mmHg)	0.986 (0.981–0.991)	<0.001	0.981 (0.978–0.984)	<0.001
Adherence	1.619 (1.367–1.917)	<0.001	1.473 (1.299–1.589)	<0.001
Cardiovascular disease (MI & coronary)	1.310 (1.066–1.609)	0.010		
Total cholesterol (per 1 mg/dL)	0.998 (0.996–0.999)	0.007	0.997 (0.996–0.999)	<0.001
Valsartan dose (0/80/160 mg)	0.726 (0.608–0.867)	<0.001	0.803 (0.720–0.894)	<0.001
HCTZ dose (0/12.5/25 mg)	0.757 (0.635–0.903)	0.002	0.683 (0.611–0.764)	<0.001
Study: INSIST^¥^			1.630 (1.173–2.265)	0.004
Study: PREVIEW^¥^	0.560 (0.431–0.728)	<0.001	0.721 (0.610–0.851)	<0.001
**Physician determinants**				
Years in practice (per 1 year)			0.988 (0.982–0.995)	<0.001

SBP: systolic blood pressure; DBP: diastolic blood pressure; HT: hypertension; HCTZ: hydrochlorothiazide; CI: confidence interval; OR: odds ratio; MI: myocardial infarct. ^¥^Reference study: EXCELLENT.

**Table 6:** Summary of determinants retained in hierarchical linear and logistic regression modeling (any occurrence of determinant).

	Diabetic					Nondiabetic				
	BP values		BP control			BP values		BP control		
	SBP	DBP	SBP	DBP	SBP/DBP	SBP	DBP	SBP	DBP	SBP/DBP
**Patient determinants**										
Demographics										
Age, per 1 year		+		+		−	+	−	+	
Blood pressure										
SBP at diagnosis of HTN, per 1 mm Hg	−		−		−	−		−		−
DBP at diagnosis of HTN, per 1 mm Hg		−		−			−		−	
Risk factors										
Renal impairment						+		−		
Cardiovascular disease (MI & coronary)	+	+	+		+	+			+	
Body mass index, per 1 kg/m^2^						−	−			
Total cholesterol, per 1 mg/dL	−	−	−	−	−	−	−	−	−	−
Valsartan dose (0/80/160 mg)	−	−	−		−	−	−	−	−	−
HCTZ dose (0/12.5/25 mg)	−	−	−	−	−	−	−	−	−	−
Adherence	+	+		+	+	+		+	+	+
Concomitant antihypertensive treatment										
*β*-Blocker	−					−				
Studies										
PREVIEW	−	−	−		−			−		−
IMPROVE		+		+					+	
INSIST							+	+	+	+
**Physician determinants**										
Year in practice, per 1 year		−				−		−		−
Duration of visit of newly diagnosed HTN patients		+								
HTN patients in past year (per 1 patient)						+	+		+	
Male gender	−					−	−			

BP: blood pressure; DBP: diastolic blood pressure; SBP: systolic blood pressure; HCTZ: hydrochlorothiazide; HTN: hypertension. Negative impact is denoted by a minus (−) sign: increases BP levels and decreases odds of BP control. Positive impact is denoted by a plus (+) sign: decreases BP levels and increases odds of BP control.

## References

[1] Ferrannini E., Cushman W. C. (2012). Diabetes and hypertension: the bad companions. *The Lancet*.

[2] Kannel W. B., Neaton J. D., Wentworth D. (1986). Overall and coronary heart disease mortality rates in relation to major risk factors in 325,348 men screened for the MRFIT. *American Heart Journal*.

[3] Stamler J., Vaccaro O., Neaton J. D., Wentworth D. (1993). Diabetes, other risk factors, and 12-yr cardiovascular mortality for men screened in the multiple risk factor intervention trial. *Diabetes Care*.

[4] Adler A. I., Stratton I. M., Neil H. A. W. (2000). Association of systolic blood pressure with macrovascular and microvascular complications of type 2 diabetes (UKPDS 36): prospective observational study. *British Medical Journal*.

[5] Mancia G., Fagard R., Narkiewicz K. (2013). 2013 ESH/ESC guidelines for the management of arterial hypertension: the Task Force for the Management of Arterial Hypertension of the European Society of Hypertension (ESH) and of the European Society of Cardiology (ESC). *European Heart Journal*.

[6] James P. A., Oparil S., Carter B. L. (2014). 2014 Evidence-based guideline for the management of high blood pressure in adults: report from the panel members appointed to the Eighth Joint National Committee (JNC 8). *Journal of the American Medical Association*.

[7] Abraham I., MacDonald K., Hermans C. (2011). Real-world effectiveness of valsartan on hypertension and total cardiovascular risk: review and implications of a translational research program. *Vascular Health and Risk Management*.

[8] Villa L., Sun D., Denhaerynck K. (2015). Predicting blood pressure outcomes using single-item physician-administered measures: a retrospective pooled analysis of observational studies in Belgium. *British Journal of General Practice*.

[9] Twisk J. W. R. (2006). *Applied Multilevel Analysis: A Practical Guide for Medical Researchers*.

[10] Abraham I., Van Camp Y., Villa L. (2013). Hierarchical modeling of patient and physician determinants of blood pressure outcomes in adherent vs nonadherent hypertensive patients: Pooled analysis of 6 studies with 14,646 evaluable patients. *Journal of Clinical Hypertension*.

[11] UK Prospective Diabetes Study Group (1999). Tight blood pressure control and risk of macrovascular and microvascular complications in type 2 diabetes: UKPDS 38. *British Medical Journal*.

[12] UK Prospective Diabetes Study Group . (1998). Efficacy of atenolol and captopril in reducing risk of macrovascular and microvascular complications in type 2 diabetes: UKPDS 39. *BMJ*.

[13] Hansson L., Zanchetti A., Carruthers S. G. (1998). Effects of intensive blood-pressure lowering and low-dose aspirin in patients with hypertension: principal results of the Hypertension Optimal Treatment (HOT) randomised trial. *Lancet*.

[14] Zanchetti A., Hansson L., Clement D. (2003). Benefits and risks of more intensive blood pressure lowering in hypertensive patients of the HOT study with different risk profiles: does a J-shaped curve exist in smokers?. *Journal of Hypertension*.

[15] Abraham I., Kurdi S., MacDonald K. (2017). The hypertension, diabetes and chronic kidney disease triangle in Arab countries. *Journal of Human Hypertension*.

[16] Gouni-Berthold I., Berthold H. K. (2012). Role of physician gender in drug therapy. *Handbook of Experimental Pharmacology*.

[17] Verpooten G. A., Aerts A., Coen N. (2011). Antihypertensive effectiveness of aliskiren for the 'real-world' management of hypertension: multilevel modelling of 180-day blood pressure outcomes (the Belgian DRIVER Study). *International Journal of Clinical Practice*.

[18] Choudhry N. K., Fletcher R. H., Soumerai S. B. (2005). Systematic review: the relationship between clinical experience and quality of health care. *Annals of Internal Medicine*.

